# An upper limit of Cr-doping level to Retain Zero-strain Characteristics of Li_4_Ti_5_O_12_ Anode Material for Li-ion Batteries

**DOI:** 10.1038/srep43335

**Published:** 2017-02-24

**Authors:** Hannah Song, Tae-Gyung Jeong, Su-Won Yun, Eun-Kyung Lee, Shin-Ae Park, Yong-Tae Kim

**Affiliations:** 1 Department of Energy Systems, Pusan National University, Busan 609-735, Republic of Korea

## Abstract

Since Li_4_Ti_5_O_12_ as a promising anode material in lithium-ion batteries (LIBs) has a poor rate performance due to low electronic conductivity, a doping of Li_4_Ti_5_O_12_ with heterogeneous atoms has been considered to overcome this problem. Herein, we report that there is an upper limit of doping level to maintain the zero strain characteristics of Li_4_Ti_5_O_12_ lattice during charge/discharge process. By using synchrotron studies, it was revealed that the Li^+^ diffusivity was maximized at a certain doping level for which the conductivity was markedly increased with maintaining the zero strain characteristics. However, with more doses of dopants over the upper limit, the lattice shrank and therefore the Li^+^ diffusivity decreased, although the electronic conductivity was further increased in comparison with the optimal doping level.

Lithium-ion batteries are a commercial success, but there are safety concerns about the carbon-based negative-electrode materials used in them^1^,^2^. Because their operating potential is relatively low and close to that of lithium metal, dendrites form on the surface of the carbon-based negative electrodes causing a short circuit, especially at high rates^3^,^4^. Cubic spinel Li_4_Ti_5_O_12_ has attracted much attention as a promising anode material for lithium-ion batteries because of its relatively high potential above 1.5 V (vs. Li), which imparts safety and stability to the battery. However, as an insulator, Li_4_Ti_5_O_12_ shows poor rate performance because of its low electronic and ionic conductivities^5^,^6^. To overcome this problem, doping a heterogeneous atom is an effective way to enhance the rate performance by changing the insulating characteristics^7^. Various transition metals such as Mg, Cr, Mn, Ta, Y, and Mo have been used as a dopant in Li_4_Ti_5_O_12_, enhancing its rate performance^3^,^8^,^9^,^10^,^11^,^12^,^13^,^14^,^15^. Some have attributed the enhanced rate performance to the increased electronic conductivity upon doping. However, others have reported that enhanced electronic conductivity does not guarantee enhanced rate performance, probably because of the decrease in Ti^4+^, which is the sole atom that participates in the redox reaction^8^,^15^. However, because only 40% of Ti^4+^ is reduced to Ti^3+^ in the charge process (Li_4_Ti_5_O_12_ → Li_7_Ti_5_O_12_), some other factor must affect the rate performance of Li_4_Ti_5_O_12_. Lithium-ion diffusivity and electronic conductivity are the main factors that play a role in high rate performance, and many researchers have tried to synthesize nanoscale particles into various shapes to shorten the lithium-ion pathways^16^,^17^,^18^.

Under doping of a heterogeneous atoms, changes in the lattice structures in Li_4_Ti_5_O_12_ can occur because bond lengths or bonding symmetry can change around the heterogeneous dopant. Those structural changes could affect the diffusion rate of lithium ions into the lattice. We studied how those structural changes affect the lithium-ion diffusivity and rate capability of Li_4_Ti_5_O_12_. Then, we found a lattice structure requirement needed for the zero-strain Li_4_Ti_5_O_12_.

## Results and Discussion

**Figure 1:**
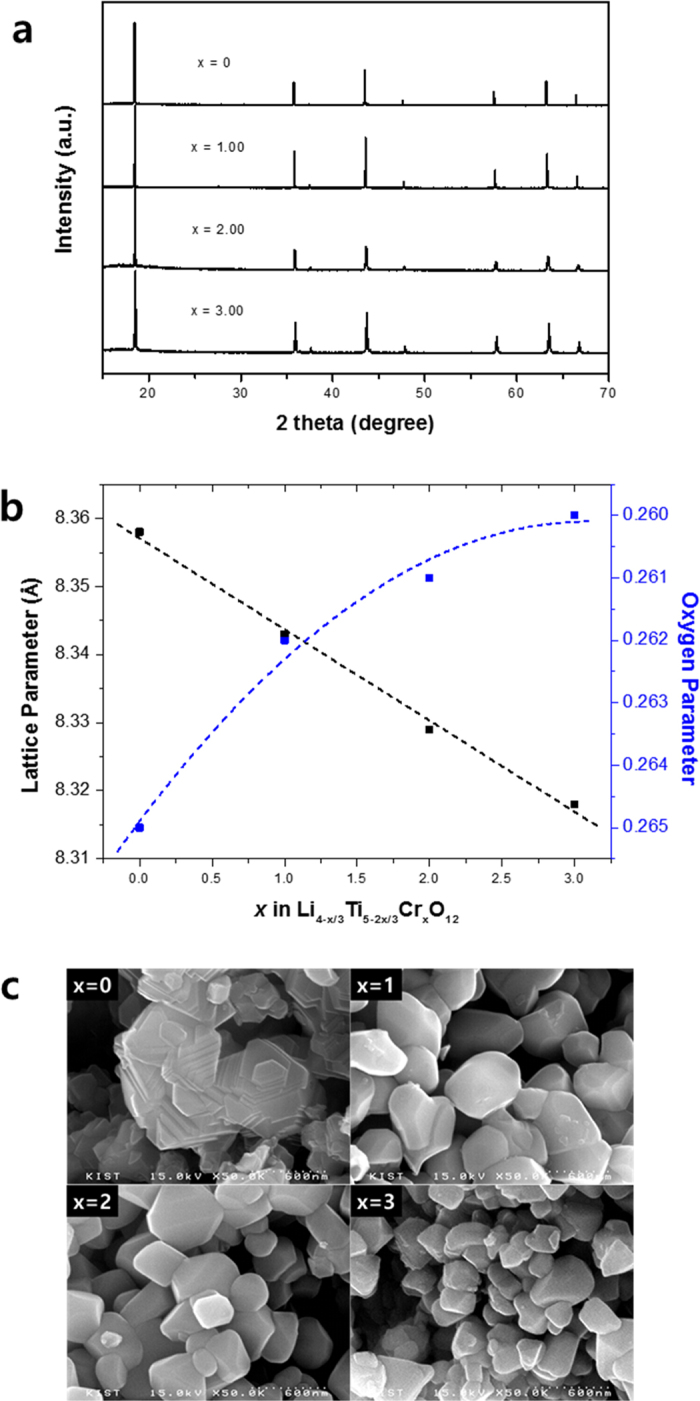
(**a**) XRD patterns (**b**) lattice parameters and oxygen parameters, and (**c**) SEM images of Li_4−*x*/3_Ti_5−2*x*/3_Cr_*x*_O_12_ (*x* = 0, 1, 2, 3).

Cr^3+^ is the dopant most used in Li_4_Ti_5_O_12_, because its high octahedral site stabilization energy (OSSE) makes it possible to substitute it for 50% of the atoms positioned at 16*d* sites^9^,^19^. In a manner similar to that used in our previous studies, we prepared Li_4−*x*/3_Ti_5−2*x*/3_Cr_*x*_O_12_ (*x* = 0, 1, 2, 3) by replacing one lithium ion and two titanium ions at the octahedral sites with three chromium ions (Li^++^ 2Ti^4+^ → 3Cr^3+^). Figure 1a presents the X-ray diffraction (XRD) patterns of the synthesized samples. All the patterns match well with the cubic spinel structure without any side peaks. When the amount of the dopant Cr^3+^ increased, a significant change in the lattice structure was observed using Rietveld refinement. Figure 1b presents the lattice parameter of Li_4_Ti_5−2*x*_Cr_*x*_O_12_, which decreased linearly as the doping amount increased. This was expected because the average ionic radii at 16*d* octahedral sites decreased when one Li^+^ (0.76 Å) and two Ti^4+^ (0.61 Å) were replaced by three Cr^3+^ (0.62 Å). Figure 1b also shows the variation in the fractional coefficient of oxygen ions in the lattice. In general, the oxygen parameter decreased when near 0.25, which is the ideal value for oxygen ions. The results imply that distortion of the lattice structure decreased with an increase in the doping amount of Cr^3+^.

**Figure 2:**
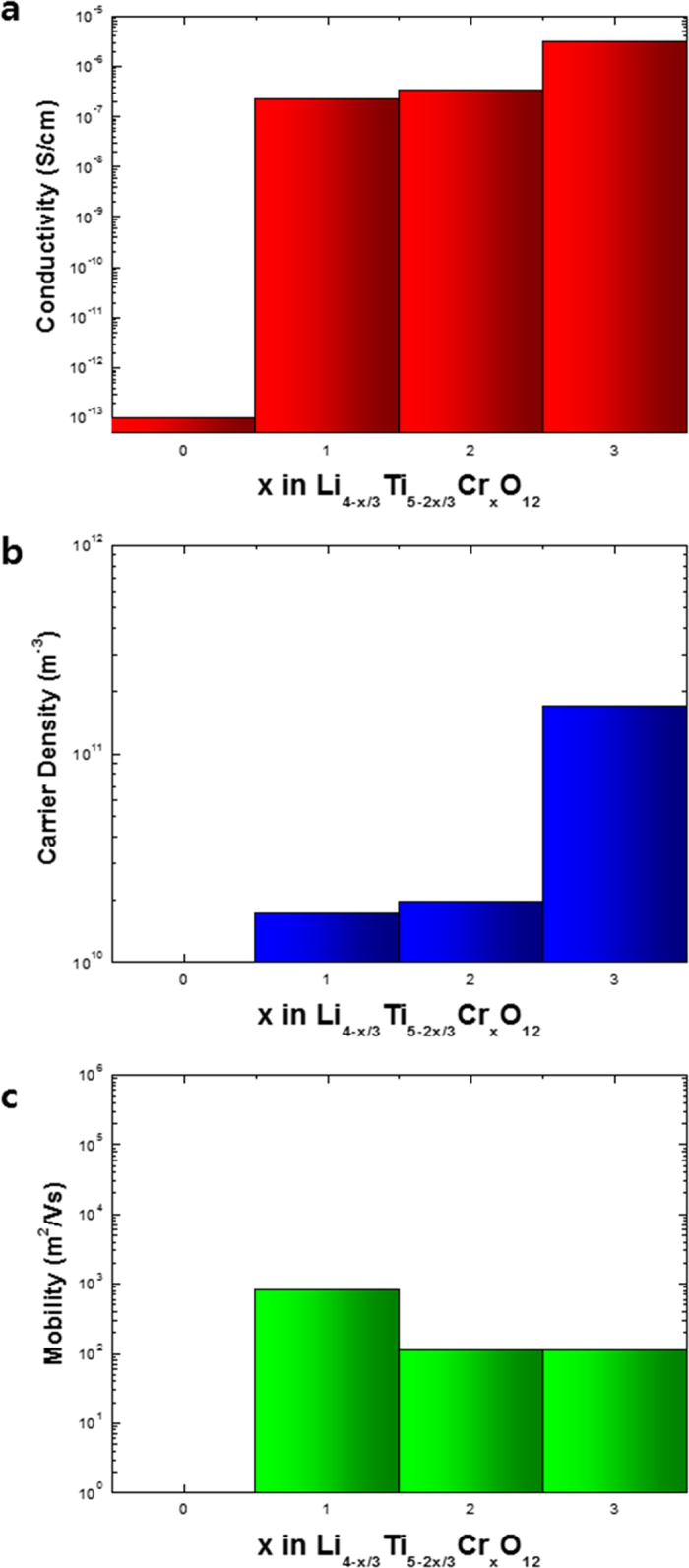
Electronic conductivity, Carrier density and Mobility of Li_4−x/3_Ti_5−2x/3_Cr_x_O_12_ (x = 0, 1, 2, 3).

Electronic conductivity of the synthesized samples was measured using the van der Pauw method. A coin type pellet of active materials was fabricated by using the laboratory hydraulic press with the maximum pressure of 25 ton. Au paste was used to form the electrode at the position of cardinal points and Au wire was used to connect the measurement port of Hall effect measurement system. The electronic conductivity of bare Li_4_Ti_5_O_12_ could not be measured because it was extremely low (<10^−13^ S/cm) and out of measurement range. As seen in Fig. 2, the electronic conductivity of Li_4_Ti_5_O_12_ increased to about 10^−7^ S/cm under doping with Cr^3+^; this is six orders of magnitude greater than that of bare Li_4_Ti_5_O_12_. The electronic conductivity was markedly enhanced with the increase of doping level, because the carrier density was increased by the extra electrons donated by Cr^3+^ (Fig. 2).

**Figure 3:**
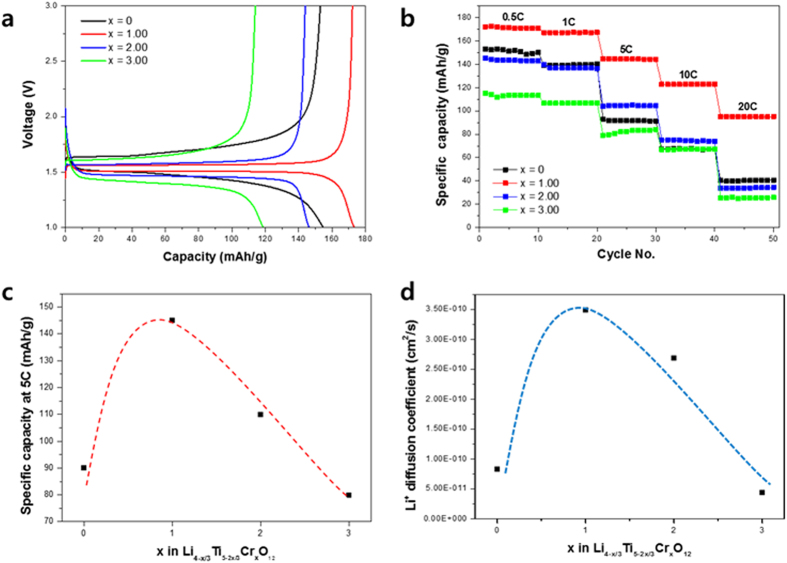
(**a**) Charge–discharge curves at 0.5 C (**b**) rate capability (**c**) specific capacity at 5 C, and (**d**) Li^+^ diffusivity of Li_4−*x*/3_Ti_5−2*x*/3_Cr_*x*_O_12_ (*x* = 0, 1, 2, 3).

In general, the enhanced electronic conductivity results in enhanced rate performance of Li_4_Ti_5_O_12_. To understand the effect of doping on electrochemical performance, we performed battery tests at various C-rates. Figure 3a shows representative charge–discharge curves for all samples at 0.5 C. The voltage difference between the charge and discharge curves decreased with Cr^3+^ doping, indicating that the voltage polarization decreased upon doping. However, the polarization increased with the Cr^3+^ doping level over *x* = 1. The specific capacity of each sample was inversely proportional to the voltage difference between the charge and discharge curves. Figure 3b shows the rate capability of all samples. The Li_4−*x*/3_Ti_5−2*x*/3_Cr_*x*_O_12_ (*x* = 1) had the highest rate capability because it had the smallest voltage difference between the charge and discharge curves. Also, the Li_4−*x*/3_Ti_5−2*x*/3_Cr_*x*_O_12_ (*x* = 1) exhibited good cycle performance at 1 C in Fig. S2. However, Fig. 3b and c show that the rate capability decreased with increasing the doping level of Cr^3+^ over *x* = 1 despite the increased electronic conductivity. Therefore, using the galvanostatic intermittent titration technique (GITT), we measured Li^+^ diffusivity, which is one of the main factors that affect the rate capability. Figure 3d shows the Li^+^ diffusivity of Li_4−*x*/3_Ti_5−2*x*/3_Cr_*x*_O_12_ with increasing doping amount of Cr^3+^. While the electronic conductivity increased linearly in Fig. 2, the Li^+^ diffusivity showed a volcano-type variation, with a peak at *x* = 1 for Li_4−*x*/3_Ti_5−2*x*/3_Cr_*x*_O_12_, and decreased linearly when the doping amount was greater than *x* = 1, which is similar to the variation of specific capacity at a high C-rate seen in Fig. 3c. This result implies that Li^+^ diffusivity more than electronic conductivity affects the rate capability with a linear increase. The highest Li^+^ diffusivity occurs when *x* = 1 because of the decrease in structural disorder caused by the swing of oxygen ions in the lattice. However, when the doping amount of Cr^3+^ was increased over *x* = 1, the Li^+^ diffusivity linearly decreased despite the increased structural order.

**Figure 4:**
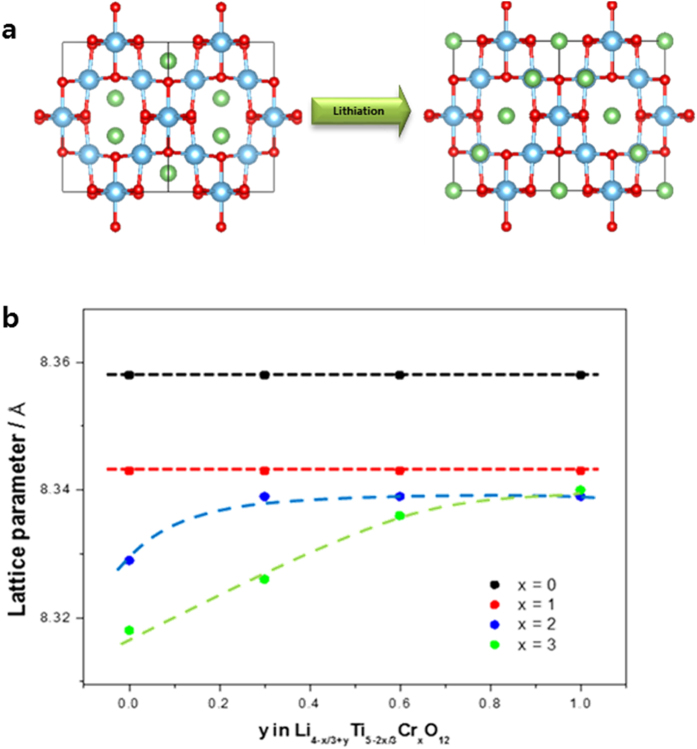
(**a**) Lattice structural changes of Li_4_Ti_5_O_12_ anode materials during lithiation showing the zero-strain characteristic of them. (**b**) Changes in the lattice dimension of Li_4−*x*/3+*y*_Ti_5−2*x*/3_Cr_*x*_O_12_ as a function of *y*.

**Figure 5:**
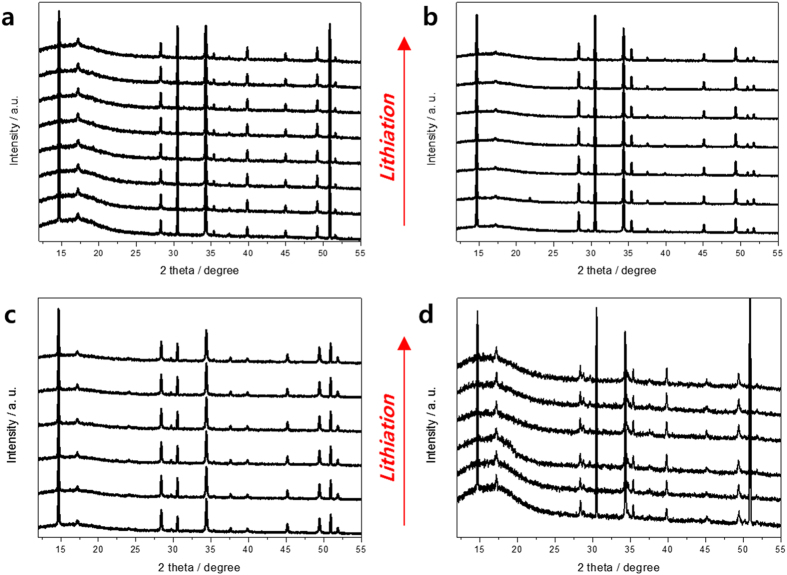
*In-situ* XRD patterns of Li_4−x/3_Ti_5−2x/3_Cr_x_O_12_ (**a**) x = 0 (**b**) x = 1 (**c**) x = 2, and (**d**) x = 3 during lithiation process.

To understand the volcano-type variation of the Li^+^ diffusivity, the particle size of all synthesized samples was observed using scanning electron microscopy (SEM) (Fig. 1c). The particle size generally decreased when the doping amount increased, which did not seem to affect the volcano-type variation of Li^+^ diffusivity. Also, Cr ions in the powder were evenly distributed as shown in Fig. S1. Therefore, we carried out *in situ* XRD measurements of all samples to identify changes to the lattice structure when Li^+^ is inserted into the lattice. Figure 4b shows changes in the lattice dimension as a function of *y* in Li_4−*x*/3+3*y*_Ti_5−2*x*/3_Cr_*x*_O_12_ (the *in situ* XRD patterns of each sample are shown in Fig. 5). For samples of Li_4−*x*/3_Ti_5−2*x*/3_Cr_*x*_O_12_ when *x* = 0 and *x* = 1, there was no significant change in the lattice dimension when Li^+^ was inserted, which is the zero-strain characteristic of the cubic spinel Li_4_Ti_5_O_12_. The lattice dimensions of the anode materials in lithium-ion batteries usually expand because of the change in ionic radii of the redox species in a solid matrix. However, during the lithiation of Li_4_Ti_5_O_12_, the oxygen ions in the lattice of Li_4_Ti_5_O_12_ swing to their ideal positions, leading to its rock salt structure without distortion from the distorted spinel structure as shown in Fig. 4a. That is why Li_4_Ti_5_O_12_ has zero strain although the Ti–O bond length increases during lithiation^20^. However, zero strain was not maintained when *x* = 2 and *x* = 3. Figure 4b shows the significant change in the lattice dimension for the two samples during lithiation. When the doping amount of Cr^3+^ increased, the lattice dimensions expanded as the lithium ions were inserted into the lattice. The lattice of the Li_4−*x*/3_Ti_5−2*x*/3_Cr_*x*_O_12_ (*x* = 2) sample expanded from 8.329 to 8.339(1) Å, and the lattice of the Li_4−*x*/3_Ti_5−2*x*/3_Cr_*x*_O_12_ (*x* = 3) sample expanded from 8.318 to 8.340(1) Å. Rapid changes in the lattice dimensions of the two samples were observed at the beginning of lithiation. These changes in lattice structure could have been caused by the decrease in lattice parameter that occurs with an increase in the doping amount of Cr^3+^. If the lattice parameter of the doped Li_4_Ti_5_O_12_ is less than about 8.34 Å, the lattice dimension will expand to about 8.34 Å during lithiation. Thus, the zero-strain characteristics of Li_4_Ti_5_O_12_ is retained when the lattice parameter is larger than a specific size, and if the doped Li_4_Ti_5_O_12_ loses the zero-strain characteristic because of a smaller lattice parameter, the Li^+^ diffusivity would decrease, resulting in the deterioration of electrochemical performance. Therefore, the changes in the lattice structure and the improvement in electronic conductivity upon doping should enhance the electrochemical performance of Li_4_Ti_5_O_12_.

In conclusion, we studied how the changes in the lattice structure of Li_4_Ti_5_O_12_ upon doping a heterogeneous atom affect its electrochemical properties. Unless the lattice parameter is larger than a specific size after doping, Li_4_Ti_5_O_12_ would lose its zero-strain characteristic because the lattice dimensions would expand during lithiation. Those structural changes cause the deterioration of the diffusion of lithium ions into the lattice, which results in the deterioration of rate capability. Therefore, to design Li_4_Ti_5_O_12_ with a high rate capability by doping a heterogeneous atom, the kind of dopant and the amount of doping should be controlled keeping in mind the changes in the lattice structure that occur.

## Methods

### Synthesis

Li_4−*x*/3_Ti_5−2*x*/3_Cr_*x*_O_12_ (*x* = 0, 1, 2, 3) was synthesized by dissolving LiOH∙H_2_O in water and adding TiO_2_ and Cr(NO_3_)_3_∙9H_2_O to the solution with the appropriate molar ratios. After ball-milling for 2 h at 50 Hz, the solution was evaporated and dried in a vacuum oven at 80 °C for 24 h. Then, the ground powder was annealed at 800 °C for 12 h in air at a heating rate of 5 °C/min.

### Characterization

Electrochemical tests were performed using 2032 coin cells assembled with working electrodes coated on Cu foil at a mass ratio of active material:acetylene black:PVDF of 80:10:10 and reference/counter electrodes made of Li metal on a Cu mesh. The mass loading of active material was about 0.06 ~ 0.062 mg/mm^2^. LiPF6 (1 M) in ethylene carbonate and dimethyl carbonate (DMC) (1:1 v/v) were used as the electrolyte. A polypropylene membrane was used as a separator. The coin cells were assembled in a glove box filled with Ar gas, and all electrochemical tests were carried out using a battery cycler (WC3000S, WonATech) at different C-rates in the voltage range of 1.0–3.0 V (vs. Li). The diffusion coefficients of the Li ions in all samples were measured using GITT with current application of 30 s^21^. High-resolution powder diffraction and *in situ* XRD patterns of the synthesized powders were measured with the 9B HRPD and 3D X-ray scattering (XRS) beamlines at the Pohang Light Source (PLS) with a wavelength of 1.5495 and 1.24 Å, respectively.

## Additional Information

**How to cite this article:** Song, H. *et al*. An upper limit of Cr-doping level to Retain Zero-strain Characteristics of Li_4_Ti_5_O_12_ Anode Material for Li-ion Batteries. *Sci. Rep.* **7**, 43335; doi: 10.1038/srep43335 (2017).

**Publisher's note:** Springer Nature remains neutral with regard to jurisdictional claims in published maps and institutional affiliations.

## Supplementary Material

Supplementary Dataset 1
