# Supplementary material for: An upper limit of Cr-doping level to Retain Zero-strain Characteristics of Li4Ti5O12 Anode Material for Li-ion Batteries
Source: Sci Rep. 2017 Feb 24;7:43335. doi: 10.1038/srep43335 (PMC5324106; doi:10.1038/srep43335)
Supplement: Supplementary Dataset 1 [file srep43335-s1.doc]

**Supplementary Information**

**An upper limit of Cr-doping level to Retain Zero-Strain Characteristics of Li_4_Ti_5_O_12_ Anode Material for Li-ion Batteries**

Hannah Song, Tae-Gyung Jeong, Su-Won Yun, Eun-Kyung Lee, Shin-Ae Park, Yong-Tae Kim^z^

*Department of Energy Systems, Pusan National University, Busan 609-735, Republic of Korea*

**Correspondence and requests for materials should be addressed to Y.-T.K. (email:* [*yongtae@pusan.ac.kr*](mailto:yongtae@pusan.ac.kr)*)*


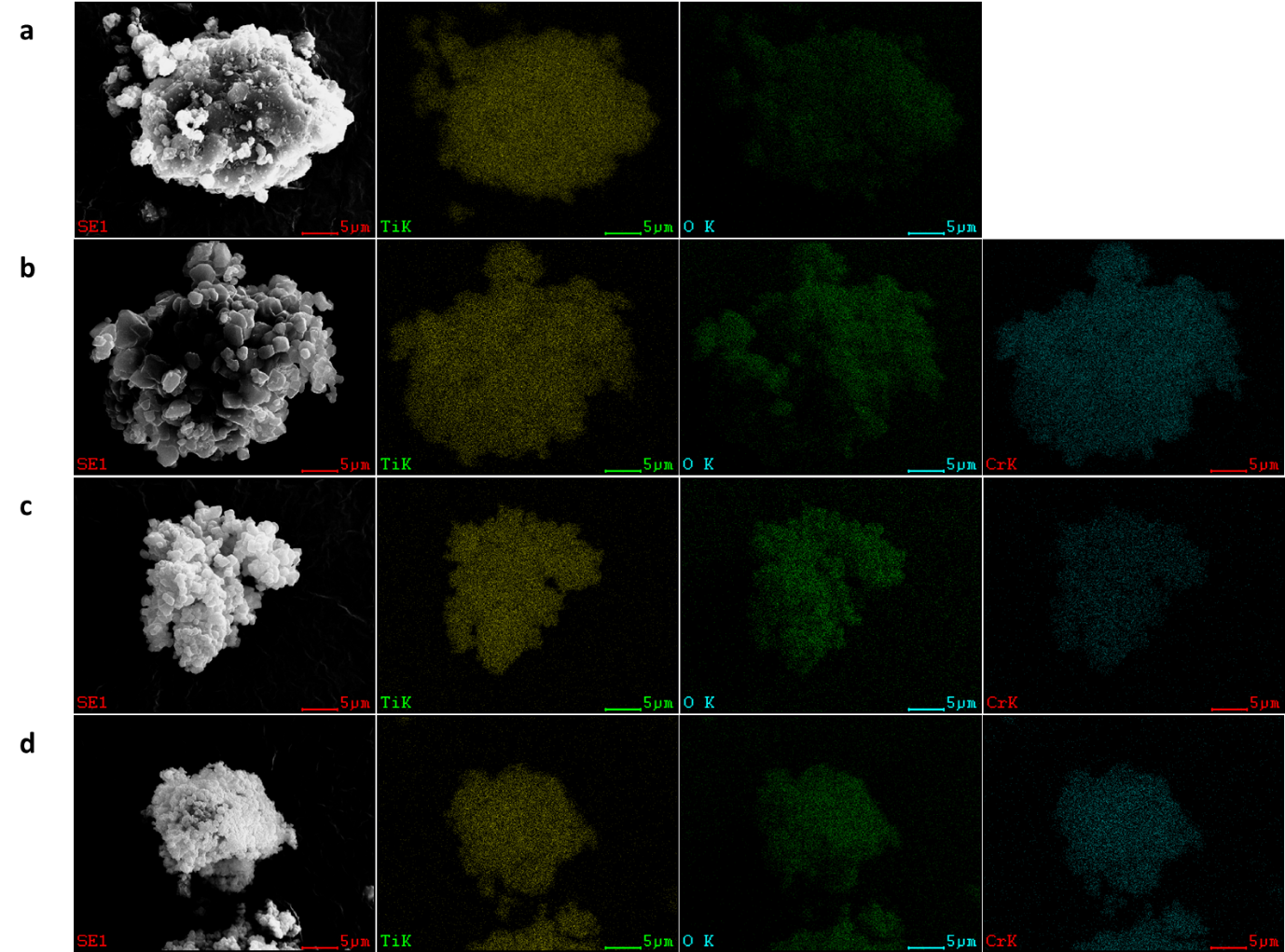


**Figure S1.** SEM-EDS images of Li_4-x/3_Ti_5-2x/3_Cr_x_O_12_ ;(a ) x=0, (b) x=1, (c) x=2, and (d) x=3

**Figure S2.** Cycle performance of Li_4-x/3_Ti_5-2x/3_Cr_x_O_12_ (x = 1) at 1 C.
